# Enhancing Drought Tolerance in *Salicornia ramosissima* Through Biofertilization with Marine Plant Growth-Promoting Bacteria (PGPB)

**DOI:** 10.3390/plants14081227

**Published:** 2025-04-16

**Authors:** Ricardo Cruz de Carvalho, João Albuquerque Carreiras, Ana Rita Matos, Isabel Caçador, Bernardo Duarte

**Affiliations:** 1 MARE—Marine and Environmental Sciences Centre & ARNET—Aquatic Research Network Associated Laboratory, Faculdade de Ciências da Universidade de Lisboa, Campo Grande, 1749-016 Lisbon, Portugalmicacador@ciencias.ulisboa.pt (I.C.); baduarte@ciencias.ulisboa.pt (B.D.); 2 CE3C—Centre for Ecology, Evolution and Environmental Changes, Faculdade de Ciências da Universidade de Lisboa, Campo Grande, 1749-016 Lisbon, Portugal; 3 BioISI—Biosystems and Integrative Sciences Institute, Departamento de Biologia Vegetal, Faculdade de Ciências da Universidade de Lisboa, Campo Grande, 1749-016 Lisbon, Portugal; armatos@ciencias.ulisboa.pt; 4 Departamento de Biologia Vegetal, Faculdade de Ciências da Universidade de Lisboa, Campo Grande, 1749-016 Lisbon, Portugal

**Keywords:** halophyte, drought stress, bioaugmentation, stress physiology

## Abstract

The duration, frequency, and intensity of drought events in the Mediterranean region pose increasing threats to conventional crop production. Consequently, eco-friendly and sustainable development approaches should aim to address future food production goals. Halophytes, such as *Salicornia ramosissima* J. Woods, represent promising cash crops for cultivation in conjunction with novel biofertilization strategies involving plant growth-promoting bacteria (PGPB). In the present study, the physiological fitness of *S. ramosissima* under various drought conditions, with and without marine PGPB inoculation, was evaluated to enhance the resilience of this cash crop halophyte under water-limited conditions. Our results indicate that PGPB inoculation significantly decreased water loss under extreme drought, with non-inoculated plants showing a water content (WC) of 59%, while in inoculated plants, the decrease in WC was lower at 77%. Furthermore, PGPB inoculation significantly enhanced the photochemistry of the plant, which maintained higher active oxygen-evolving complexes and a greater ability for complete closure of reaction centers under severe and extreme drought, thus demonstrating an improved capacity for light energy utilization in photosynthesis even under water-limited conditions. Furthermore, bioaugmented plants generally exhibited improved osmoregulation through increased yet appropriate accumulation of proline, a major osmolyte, and higher relative water content in the stem compared to the corresponding non-inoculated plants. Drought stress similarly modified the fatty acid profile in both plant groups, resulting in increased membrane stability due to reduced fluidity. However, PGPB-inoculated plants demonstrated a higher capacity for mitigation of oxidative stress, primarily through enhanced activities of superoxide dismutase, which is crucial for the scavenging of harmful reactive oxygen species (ROS). This, along with improvements in energy use and dissipation, as evidenced by photochemistry, reveals a multi-dimensional mechanism for drought tolerance in bioaugmented plants. Metabolic changes, particularly in PGPB-inoculated plants, clearly demonstrate the potential of these bacteria to be utilized in the enhancement of drought tolerance in *S. ramosissima*. Moreover, these data elucidate the complex metabolic aspects regarding photochemistry, osmoregulation, and oxidative stress that should be considered when phenotyping plants for drought tolerance, given the increasing water scarcity worldwide scenario.

## 1. Introduction

Food security constitutes a fundamental necessity in any society. The global human population is projected to reach 10 billion within the next five decades, generating an unprecedented demand that will require a 50% increase in the yield of major food crops [1]. Nevertheless, contemporary adaptive agricultural practices are inadequate to address the consequences of global climate change, which is resulting in diminished soil fertility [2]. In this context, changing climatic conditions are generating novel challenges through extreme heat stress, salinization, and an increased frequency and intensity of droughts in agriculture [2]. Over the past two decades, drought episodes have intensified in Europe, particularly in the Mediterranean region. Average summer temperatures have increased by 2.3 °C, and precipitation is projected to decrease by an additional 30% by 2050 [3,4]. Prolonged and more severe droughts in regions already experiencing water scarcity, coupled with unpredictable precipitation extremes, may present significant challenges to crop production in the Mediterranean region (e.g., an 11% loss in maize yield and 12% in wheat yield), with countries such as Portugal, Spain, Greece, and Bulgaria potentially experiencing crop losses of up to 80% [3,4]. Consequently, there is an urgent and increasing necessity for sustainable and regenerative agricultural practices that can maintain crop production despite limited water availability. This escalating water scarcity, further exacerbated by climate change and unsustainable water management practices, poses a significant threat to global food security [5]. Consequently, it is imperative to conduct further investigations into the utilization of alternative crops, such as halophytes, and their associated microbiomes to develop efficacious cultivation strategies that ensure future food requirements and agricultural resilience in the context of climate change scenarios [6].

Halophytes are an important group of plants, recognized for their nutritional value [7] and agroecological benefits [8]. These remarkable plants exhibit high tolerance not only to various physiological constraints, including soil salinization [9,10,11], elevated CO_2_ levels [12,13], and challenging thermal conditions [14,15,16], but also to drought stress [17,18,19]. Nevertheless, despite their resilience, halophytes may exhibit reduced biomass production under extreme environmental conditions [20]. Therefore, the development of nature-based solutions (NBS) is essential to assist these valuable and resilient crops in overcoming physiological limitations.

The rhizosphere, defined as the region of soil influenced by plant root activity, harbors diverse bacterial communities (rhizobacteria) that interact with host plants [21]. These interactions range from neutral (commensal bacteria), to negative (phytopathogenic bacteria), and positive (plant growth-promoting bacteria, or PGPB) [21,22].

The drought response of plants is a complex physiological process involving multiple phytohormones, among which abscisic acid (ABA), ethylene, jasmonic acid, and salicylic acid are the primary regulatory factors [23]. This response can be further modulated by PGPB, based on their effects on hormone levels, influencing stress responses and root development through various mechanisms [24,25]. For example, PGPB may induce increased levels of ABA, to initiate stomatal closure, reducing water loss [26,27], or suppress the production and level of ethylene, preventing stress-related growth inhibition [28]. In addition to these hormonal modulations, PGPB produce volatile organic compounds, such as 2,3-butanediol and acetoin, which, by inducing the mitogen-activated protein kinase (MAPK) cascade in the plant cell, elicit the expression of genes involved in drought tolerance [29,30]. These include genes encoding antioxidant enzymes (such as superoxide dismutase, catalase, and peroxidase), those associated with stomatal closure, and those responsible for the accumulation of protective osmolytes, as well as stress-responsive proteins such as dehydrins and late embryogenesis abundant (LEA) proteins, which play a crucial role in maintaining cellular integrity and osmotic balance [31,32,33,34].

Halophyte plants demonstrate the capacity to flourish in saline environments and exhibit notable adaptations to osmotic and oxidative stresses [8]. Although halophytes exhibit higher levels of drought and salt tolerance compared to common crops, their growth rates are adversely affected by both stresses, which frequently induce biochemical and morphological changes, resulting in significant alterations in metabolite profiles [35,36]. Nevertheless, drought, salinization, extreme temperature, and nutrient limitation associated with semi-arid environments could be mitigated by the application of PGPB [37,38]. Halophytes have been demonstrated to influence their rhizosphere bacterial community structure, with research indicating that the rhizospheres of these species tend to harbor higher bacterial abundance and diversity compared to the surrounding bulk sediments [39,40,41,42]. Several PGPBs isolated from halophyte rhizospheres have demonstrated beneficial effects on mitigating drought and saline stress in both halophytes and conventional crops [43,44,45,46,47,48,49,50,51,52]. These PGPBs enhance nutrient acquisition through mechanisms such as nitrogen fixation, nutrient solubilization, and siderophore production for iron acquisition [22]. Despite the potential of PGPB, attempts at targeted “rhizosphere engineering” involving selective inoculations for improved crop production remain notably limited [53]. However, the selection of the most efficacious inoculants necessitates a more comprehensive understanding of the mechanistic relationships between PGPB traits and plant responses. Nonetheless, PGPB bioaugmentation of halophytes presents a promising and cost-effective NBS for enhancing agricultural production under adverse conditions [54]. Previous works have highlighted the potential use of marine PGPB to enhance plant stress tolerance and optimize crop cultivation, namely in strawberry [55,56], tomato [57], rice [58], legumes [59], grapevine [43] and Swiss chard [46], but also to enhance halophyte fitness under varying stressful conditions [11,60,61,62,63,64].

Cash crop halophytes, comprising approximately 2500 species worldwide, have garnered attention for their agricultural potential [65]. Among these species, *Salicornia ramosissima* J. Woods (Amaranthaceae), which is commonly referred to as pickleweed, glasswort, sea beans, sea asparagus, crow’s foot greens, and samphire, is notable for its nutritional properties [66,67,68]. While its historical utilization as a salt source is well-documented, recent scholarly interest in sustainable food resources has brought this halophyte to the forefront of scientific inquiry [67]. The aerial parts of *Salicornia* species, previously regarded as a culinary delicacy, demonstrate a high mineral content (magnesium, potassium, and calcium), lipophilic phytochemicals, and antioxidant compounds, thus establishing its potential as an emerging functional food [48,49,68,69,70,71]. Nevertheless, similar to numerous succulent halophytes, *Salicornia* species exhibit vulnerability to drought stress, and their osmotic equilibrium is significantly compromised during high-intensity drought events [17,18,19].

In the present study, we evaluated the impact of marine PGPBs isolated from Portuguese salt marshes on the physiological fitness of *S. ramosissima* under three distinct drought conditions. This assessment involved examining the impact of a selected rhizobacterial consortium, exploiting the complementarity of plant growth-promoting traits, on the photochemical performance, temperature, proline and water content, pigment composition, fatty acid profiles, and antioxidant defenses of *S. ramosissima*. Data for these parameters were used to help elucidate the mechanisms by which the bacterial consortium could potentially alleviate drought stress experienced by *S. ramosissima*.

## 2. Material and Methods

### 2.1. Experimental Setup

*Salicornia ramosissima* J. Woods plants, in mature stage of development, were acquired from RiaFresh (Faro, Portugal), and planted in propagation trays containing 200 cm^3^ of organic commercial soil per plant. After arrival, the plants were placed in controlled-environment chambers (Aralab/Fiberoclima 18000EH, Lisbon, Portugal), under a photoperiod of a 16 h/8 h light/dark cycle, at temperatures of 25 °C during the light period and 14 °C in the dark, with a light intensity of 300 μmol m^−2^ s^−1^, and with relative humidity at 50 ± 5%. Plants were watered with distilled water in the trays every two days, maintaining permanent water availability through continuous capillarity water absorption, and acclimation was allowed for two weeks.

A rhizobacterial consortium was formulated with the objective of combining complementary plant growth-promoting activities to enhance stress resilience in *S. ramosissima*, which may have synergistic effects on plant resilience against stress (Supplementary Table S1). The following consortium of rhizobacteria were isolated from distinct halophytes inhabiting the Portuguese west coast salt marshes: *Bacillus aryabhattai* SP20 and *Stenotrophomonas rhizophila* EH7, isolated in the Aveiro coastal lagoon (coordinates 40°45′ N 8°56′ W); *Pseudomonas oryzihabitans* RL18 and *Salinicola endophyticus* EL13, obtained at the Tagus estuary, located in Lisbon (coordinates 38°46′ N 9°05′ W). These rhizobacteria had been isolated, characterized, and identified, under the context of a prior work [72].

Before root inoculation, each rhizobacterial suspension was grown separately in 250 mL Erlenmeyer flasks containing 50 mL of modified tryptic soy broth (TSB) medium supplemented with 25 g L^−1^ NaCl. Flasks were incubated in a rotary shaker for 24 h at 150 rpm and 30 °C. Cultures were then centrifuged at 5000× *g* for 10 min at room temperature and the supernatant was removed. The bacterial pellets were washed twice with sterile physiological saline solution (0.9% *w*/*v* NaCl) and adjusted to a final concentration of 10^8^ CFU mL^−1^ (OD_600_ = 1) according to the methodology described by [13]. The bacterial suspensions were subsequently combined in equal proportions to produce a final consortium suspension for root inoculation. Compatibility between bacterial species was assessed through co-cultivation on solid tryptic soy agar (TSA) medium. The growth halo diameter of the co-culture was compared to that observed in single-species culture. No significant differences were detected, indicating compatibility among the bacterial species.

**Figure 1:**
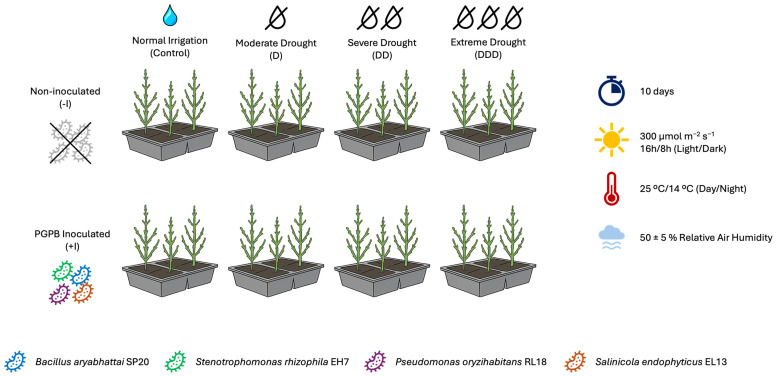
Experimental setup of the assay with the controlled environment conditions of the *Salicornia ramosissima* J. Woods control (Control) plants and the individuals exposed to moderate (D), severe (DD), and extreme (DDD) drought conditions with (+I) and without (−I) marine plant growth-promoting bacteria (PGPB) inoculation (N = 5, average).

After acclimatization, plants in pots containing 200 cm^3^ of commercial organic soil per plant were divided into two groups for root inoculation. Plants of the first group received 50 mL of the prepared inoculant solution, which was previously diluted in distilled water to a final concentration of 10^7^ CFU mL^−1^. Plants in the second group were administered 50 mL of 0.9% *w*/*v* saline solution without bacterial inoculation. Inoculated and non-inoculated plants were irrigated daily for one week prior to the experiment. Subsequently, the plants were subjected to a 10-day assay under the following conditions: daily irrigation (control), irrigation every two days (moderate drought, D), irrigation only on the fifth day (severe drought, DD), and no irrigation throughout the 10-day experiment (extreme drought, DDD). Five replicate plants were allocated to each treatment and the phytoclimatic chambers maintained temperature, light intensity, and relative humidity conditions similar to those of their initial acclimatization (Figure 1).

### 2.2. Pulse Amplitude Modulated (PAM) Fluorometry

The PAM chlorophyll fluorescence of *S. ramosissima* was measured after 30 min dark incubation at the end of their respective exposure trials using the Customized Open FluorCam Imaging system—FC 800-D/3535-FAST (Photo System Instruments, Drásov, Czech Republic). Kautsky curves or OJIP transient measurements were performed to find the functionality of the photochemical apparatus through the chlorophyll fluorescence induction kinetics of Photosystem II (PSII). Dark-adapted plants were exposed to a saturating light intensity of 3500 µmol m^−2^ s^−1^, which led to a polyphasic rise in fluorescence (OJIP) characterized by different stages: level O when all reaction centers (RCs) are open at the onset of illumination, with no QA reduction (fluorescence intensity lasting 10 ms); O to J transient corresponding to the net photochemical reduction of QA (the stable primary electron acceptor of PSII) to Q_A_^−^ (lasting 2 ms); J to I transition resulting from all reduced states of closed RCs, including Q_A_^−^ Q_B_^−^, Q_A_ Q_B_^2−^, and Q_A_^−^ Q_B_ H_2_ (lasting 2–30 ms); and the P-step, which coincides with the maximum concentration of Q_A_^−^ Q_B_^2^, with the plastoquinol pool maximally reduced, reflecting a balance between the light incident at the PSII side and the rate of utilization of chemical (potential) energy versus the rate of heat dissipation [73].

Table 1 presents the parameters derived from the fluorometric analysis. Subsequently, photosynthetic stem samples were harvested, immediately flash-frozen in liquid N_2_, and stored at −80 °C for subsequent biochemical analysis.

### 2.3. Temperature and Water Content

Thermal imaging was performed using an infrared camera (FLIR E50bx, FLIR Systems, Inc., Wilsonville, OR, USA), with an image resolution of 320 × 240 and precision of ±0.045 °C. In all treatment combinations, five photosynthetic stems were randomly selected for analysis. A water recipient at ambient temperature positioned adjacent to the stems served as the temperature reference. The average temperature of each individual stem was calculated from captured thermal images. Subsequent image processing and analysis were conducted using FLIR Tools software (version 6.4.18039.1003, FLIR Systems, Inc., Wilsonville, OR, USA).

Specimens from each treatment group were measured for mass prior to (fresh weight, FW) and following freeze-drying (dry weight, DW). Stem water content (WC) was subsequently determined utilizing the following equation:

$$WC\left(\%\right)=\frac{\left(FW-DW\right)}{FW}\times 100$$

### 2.4. Proline Content

The proline content in photosynthetic stems was determined using the method described by [74]. Initially, the plant material underwent homogenization with sulfosalicylic acid (3%, *w*/*v*). The samples were subsequently centrifuged at 9000× *g* for 15 min at 4 °C. Following centrifugation, 2 mL of glacial acetic acid and 2 mL of acidic ninhydrin were added to 2 mL of the supernatant. The resulting mixture was incubated for 1 h at 100 °C in a dry bath, after which the reaction was terminated in an ice bath. Upon cooling, 4 mL of toluene was added to the reaction mixture and vortexed to ensure thorough mixing of the organic and aqueous phases. Following the separation of the two phases, the absorbance of the organic phase was measured at 520 nm and compared to a proline standard curve. The proline content was expressed as mg g^−1^ dry weight (DW).

### 2.5. Pigment Profile Analysis

Freeze-dried photosynthetic stem samples (approximately 200 mg) were extracted using 6 mL of pure acetone. The samples were subjected to a 2 min ultrasound bath to ensure complete cell disruption. Extraction was conducted in darkness at −20 °C for 24 h. Following extraction, the samples were centrifuged at 4000× *g* for 15 min at 4 °C. Subsequently, the supernatants were scanned from 350 to 750 nm in 1 nm increments using a Shimadzu UV/VIS UV1601 Spectrophotometer with a double beam (Shimadzu Corporation, Kyoto, Japan). Sample absorbance spectra were analyzed using the Gauss-peak spectra (GPS) method fitting library, implemented in the SigmaPlot Systat 13.2 Software (Systat Software Inc., San Jose, CA, USA) [75]. This method facilitated pigment identification and quantification from the sample absorbance spectrum, thereby enabling the determination of the pigment profile in stems, including chlorophyll *a*, chlorophyll *b*, pheophytin *a*, antheraxanthin, β-carotene, lutein, violaxanthin, and zeaxanthin.

For a more comprehensive assessment of light harvesting and photoprotection mechanisms, we calculated the de-epoxidation state (DES) as follows:

$$DES=\frac{\left(\left[Antheraxanthin\right]+\left[Zeaxanthin\right]\right)}{\left(\left[Violaxanthin\right]+\left[Antheraxanthin\right]+\left[Zeaxanthin\right]\right)}$$

Anthocyanins were quantified in freeze-dried samples. The samples were subjected to grinding in a mixture of 99% methanol and 1% HCl, followed by thorough disruption of the stem material via ultrasound bath at low temperature for 2 min. The extraction process was conducted under dark conditions at −20 °C for 24 h, subsequently followed by centrifugation at 4000× *g* for 15 min at 4 °C, and measurement of the extract’s absorbance at 530 nm [76].

### 2.6. Fatty Acid Analysis

Fatty acid analysis in the photosynthetic stem was determined as described by [19]. Direct transesterification of the photosynthetic stem samples with freshly prepared methanol–sulfuric acid (97.5:2.5, *v*/*v*) was conducted in tubes placed in a bath at 70 °C for 1 h. The reaction media also contained heptadecanoic acid (C17:0), which served as an internal standard. Subsequently, the resulting fatty acid methyl esters (FAMEs) were initially extracted with petroleum ether, then dried under a stream of N_2_ and resuspended in hexane. The reconstituted FAMEs were analyzed using a PerkinElmer Clarus^®^ 590 GC gas chromatograph (PerkinElmer, Waltham, MA, USA) equipped with a hydrogen flame ionization detector set at 300 °C. One microliter of the FAME solution was injected into the system (injector temperature = 270 °C, split ratio = 50). The FAMEs were then separated in a fused-silica capillary column (50 m × 0.25 mm; WCOT Fused Silica, CP-Sil 88 for FAME; Varian, Palo Alto, CA, USA) at constant nitrogen flow (2.0 mL min^−1^) with the column oven set at 190 °C. Fatty acid identification was achieved through comparison with the retention times of the standards (Sigma-Aldrich, St. Louis, MO, USA). Chromatograms were analyzed using the peak surface method in the TotalChrom^®^ software (version 6.3.3.069, PerkinElmer, Waltham, MA, USA).

### 2.7. Oxidative Stress Biomarkers

The enzyme extracts of the samples were prepared according to the method of [77]. Plant material was homogenized with 4 mL of 50 mM sodium phosphate buffer, pH 7.6, containing 0.1 mM Na-EDTA, and maintained at 4 °C. The homogenate was centrifuged at 9000× *g* for 20 min at 4 °C. The resultant supernatant was preserved on ice for subsequent enzymatic and protein analysis.

The enzymatic activity measurements of catalase (CAT, EC 1.11.1.6), ascorbate peroxidase (APx, EC 1.11.1.11), guaiacol peroxidase (GPx, EC 1.11.1.7), and superoxide dismutase (SOD, EC 1.15.1.1) were conducted utilizing a dual-beam spectrophotometer (Shimadzu UV/VIS UV1601 Spectrophotometer, Shimadzu Corporation, Kyoto, Japan) with quartz cuvettes. Catalase activity was assayed according to the method of [78] based on following the disappearance of hydrogen peroxide (H_2_O_2_) by the decrease in absorption at 240 nm (ε = 39.4 mM^−1^ cm^−1^). Ascorbate peroxidase activity was measured by the method of [77] by following the oxidation of ascorbate by the decrease in absorbance at 290 nm (ε = 2.8 mM^−1^ cm^−1^). Guaiacol peroxidase activity was assayed using the method of [79] by monitoring the increase in absorbance at 470 nm due to guaiacol oxidation products (ε = 26.6 mM^−1^ cm^−1^). Superoxide dismutase activity was quantified based on the method by [80] which estimates the rate of pyrogallol oxidation at 325 nm. The Bradford method was used for protein quantification [81].

Lipid peroxidation in membranes was performed in samples by determining the thiobarbituric acid reactive substances (TBARS), according to the method described by [82]. A measure of 100 mg of sample was homogenized with 2 mL of a solution composed of 0.5% *w*/*v* thiobarbituric acid and 20% *w*/*v* trichloroacetic acid, both freshly prepared. After this, the samples were extracted at 95 °C in a dry bath for 30 min, and, afterwards, the reaction was stopped by transferring the samples to ice. Finally, the samples were centrifuged at 4000× *g* for 5 min at 4 °C. Absorbance of the supernatant was read at 532 and 600 nm using a Shimadzu UV-1601 spectrophotometer (Shimadzu Corporation, Kyoto, Japan). The TBARS concentration was calculated as malondialdehyde (MDA) equivalents using the MDA molar extinction coefficient (ε_MDA_ = 155 mM^−1^ cm^−1^):

$${TBARS}_{\left(MDA\;equivalents\right)}(mM)=\frac{A_{532\;nm}-A_{600\;nm}}{\varepsilon_{MDA}}$$

### 2.8. Statistical Analysis

To visualize the distribution of data, box plots of each variabl"e were constructed. Subsequently, kernel density estimation was performed utilizing the “ggplot2” package [83] in R-Studio (version 1.4.1717) to visualize smoothed density curves. The dataset was assessed for normality and homogeneity of variances. Subsequently, non-parametric Kruskal–Wallis tests with a post hoc Bonferroni adjustment were conducted using the “agricolae” package [84] in R-Studio (version 1.4.1717), comparing the effects of the inoculation and drought treatments on each variable (5 biological replicates). Multivariate analysis for each dataset (Kautsky curves, pigment concentrations, fatty acid profile, and oxidative stress biomarkers) was conducted utilizing Partial Least Squares Discriminant Analysis (PLS-DA) in R-Studio (version 1.4.1717) with the “Discriminer” package [85], allowing the assessment of biochemical and biophysical characteristics, using four components based on the number of tested drought conditions (control, D, DD, and DDD) [86,87,88], and classification of the samples with respect to the drought and inoculation treatments applied. The choice of the number of components was evaluated using the Receiver Operating Characteristic (ROC) Area Under the Curve (AUC) parameter, the goodness of fit or explained variation (R^2^), and the goodness of prediction or predicted variation (Q^2^). The overall performance of the model was subsequently evaluated utilizing each dataset as classifiers.

## 3. Results

### 3.1. Primary Photochemistry

**Figure 2:**
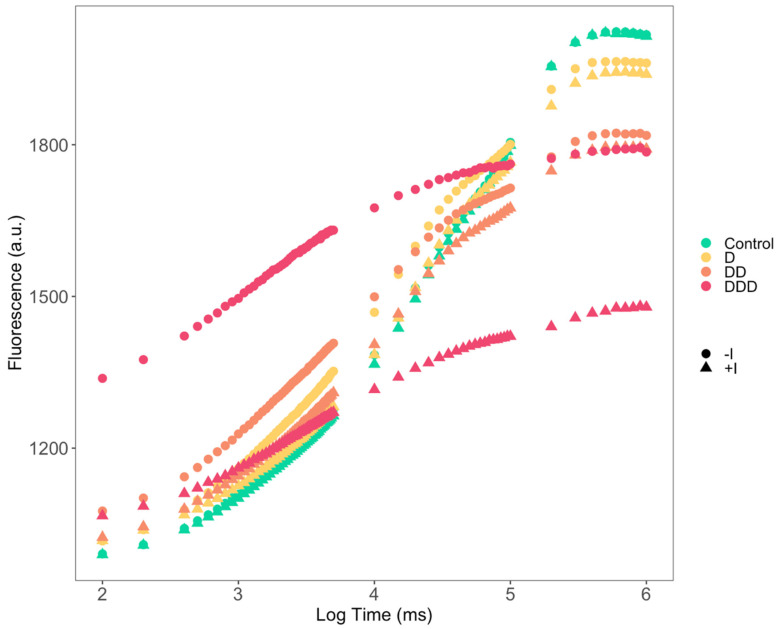
Kautsky fluorescence induction curves attained from the *Salicornia ramosissima* J. Woods control (C) plants and the individuals exposed to moderate (D), severe (DD), and extreme (DDD) drought conditions with (+I) and without (−I) marine plant growth-promoting bacteria (PGPB) inoculation (N = 5, average).

Upon examination of the Kautsky induction curves (Figure 2), significant differences were observed. The control (C) curves for plants subjected to standard hydration conditions, both inoculated (+I) and non-inoculated (−I), exhibited elevated fluorescence levels and pronounced inflection points, characteristic of a Kautsky or OJIP curve. Conversely, plants subjected to various drought stress levels demonstrated a marked reduction in fluorescence at the curve inflection points as drought severity increased. The difference between inoculated and non-inoculated plants remained minimal, except for the most severe drought scenario (DDD), where non-inoculated plants exhibited higher fluorescence values than plants supplemented with the PGPB consortium.

**Figure 3:**
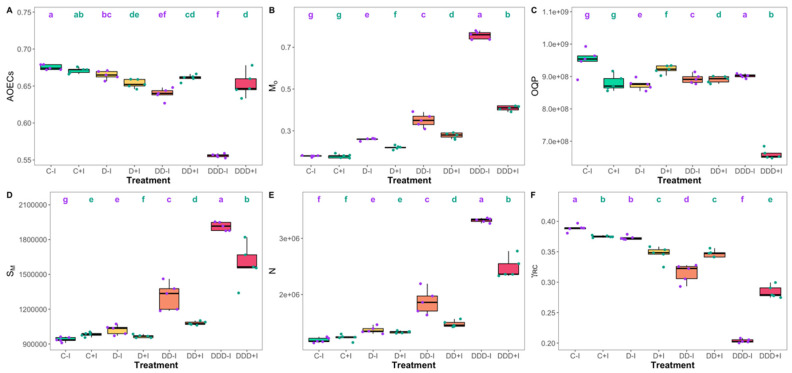
Active oxygen-evolving complexes (AOECs, (**A**)), the net rate of PSII RC closure (M_O_, (**B**)), oxidized quinone pool (OQP, (**C**)), the energy needed to close all reaction centers (S_M_, (**D**)), quinone turnover rate (N, (**E**)), and the probability that a chlorophyll molecule functions as a reaction center (γ_RC_, (**F**)) attained by the *Salicornia ramosissima* J. Woods control (C) plants and the individuals exposed to moderate (D), severe (DD), and extreme (DDD) drought conditions with (+I) and without (−I) marine plant growth-promoting bacteria (PGPB) inoculation (N = 5 per treatment, boxplots with average ± standard deviation; letters denote significant differences at *p* < 0.05).

Regarding the active oxygen-evolving complexes (AOECs, Figure 3A), no significant differences were observed between the control plant groups. However, a decrease in AOECs was observed in inoculated plants compared to non-inoculated plants under D drought stress, whereas under severe and extreme drought conditions, PGPB-inoculated plants maintained higher AOEC levels, reaching values comparable to those observed under moderate drought stress. Under control conditions, no significant differences were found in the net rate of PSII reaction center closure (M_0_, Figure 3B), which increased significantly along the drought gradient. Nevertheless, PGPB-inoculated plants maintained significantly lower M_0_ values under all drought conditions compared to non-inoculated plants. The size of the oxidized quinone pool (OQP, Figure 3C) exhibited significant variation across all treatments, with the exception of the controls. The most notable difference was the greater reduction in OQP observed in the inoculated plants subjected to extreme drought conditions. Regarding the energy required to close all reaction centers (S_M_, Figure 3D), significant increases were observed with increasing drought severity. Furthermore, inoculated plants in the control group exhibited higher S_M_ values, whereas in all other treatments, PGPB inoculation resulted in significantly lower S_M_ values compared to their non-inoculated counterparts. The quinone turnover rate (N, Figure 3E) demonstrated a similar trend to S_M_, with no significant differences observed between the plant groups under the control and moderate drought conditions. The likelihood of a chlorophyll molecule functioning as a reaction center (γ_RC_, Figure 3F) showed a clear trend of decreasing with increasing drought severity. Nevertheless, under severe and extreme drought stress, PGPB-inoculated plants exhibited significantly higher γ_RC_ values.

**Figure 4:**
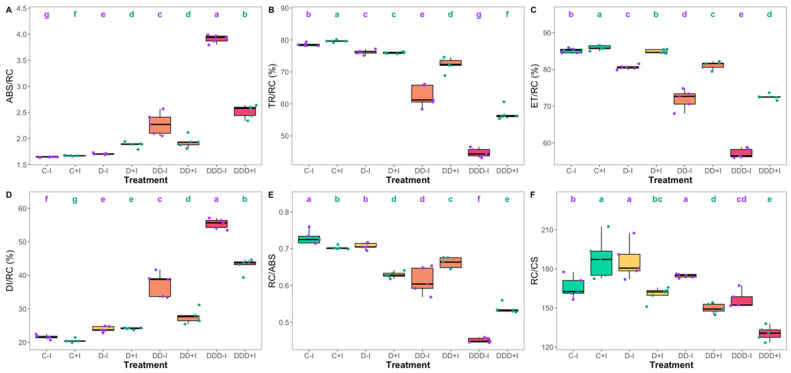
Absorbed (ABS/RC, (**A**)), trapped (TR/RC, %, (**B**)), transported (ET/RC, %, (**C**)), and dissipated (DI/RC, %, (**D**)) energy fluxes on a reaction center basis, reaction center density within the PSII antennae (RC/ABS, (**E**)), and the number of available reaction centers per stem cross-section (RC/CS, (**F**)) attained by the *Salicornia ramosissima* J. Woods control (C) plants and the individuals exposed to moderate (D), severe (DD), and extreme (DDD) drought conditions with (+I) and without (−I) marine plant growth-promoting bacteria (PGPB) inoculation (N = 5 per treatment, boxplots with average ± standard deviation; letters denote significant differences at *p* < 0.05).

With respect to energy fluxes, PGPB-biofertilized plants under control and D drought conditions exhibited an increase in absorbed energy flux (ABS/RC) compared to their non-inoculated counterparts (Figure 4A). Conversely, in plants under DD and DDD drought conditions, this trend was reversed; however, the values remained above those of the control treatments. The trapped energy flux (TR/RC) was generally higher in the inoculated plants, except for the specimens subjected to moderate drought treatment, which demonstrated no significant difference between the inoculation groups (Figure 4B). All drought treatments resulted in lower TR/RC values compared to the control condition, with the decline intensifying as drought severity increased. However, the decrease was less pronounced in inoculated plants subjected to severe and extreme drought compared to their non-inoculated counterparts. Transported energy flux (ET/RC) exhibited a similar pattern, except in the plants subjected to moderate drought treatment, where PGPB-fertilized plants demonstrated higher ET/RC values than non-inoculated plants (Figure 4C). Regarding the dissipated energy flux (DI/RC), PGPB inoculation generally led to lower values, except in the individuals subjected to moderate drought treatment, where no significant differences were observed between inoculation groups (Figure 4D). Nevertheless, similarly to what was observed for ABS/RC, drought treatments led to significant increases in DI/RC as severity increased. Examination of the reaction center density on the PSII antennae (RC/ABS) revealed that in the control and under moderate drought conditions, inoculation resulted in lower values of this parameter, whereas in individuals exposed to severe and extreme drought conditions, the opposite trend was observed (Figure 4E). Furthermore, while the number of available reaction centers per stem cross-section (RC/CS) increased in PGPB-inoculated plants under control conditions, in all drought scenarios, inoculation led to a reduction in this value when compared to the value observed in non-inoculated plants (Figure 4F).

### 3.2. Stem Temperature, Water Content, and Proline

**Figure 5:**
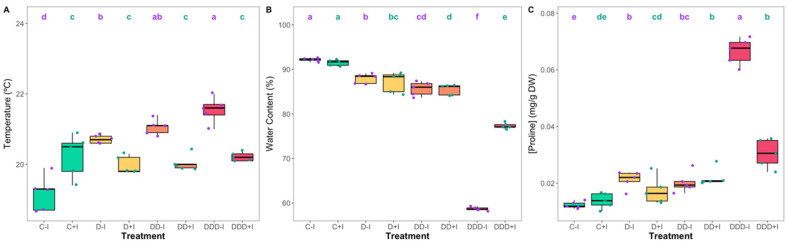
Temperature (**A**), water content (**B**), and proline concentration (**C**) measured in the *Salicornia ramosissima* J. Woods control (C) plants and the individuals exposed to moderate (D), severe (DD), and extreme (DDD) drought conditions with (+I) and without (−I) marine plant growth-promoting bacteria (PGPB) inoculation (N = 5 per treatment, boxplots with average ± standard deviation; letters denote significant differences at *p* < 0.05).

All treatments exhibited an increase in the temperature of the photosynthetic stems compared to non-inoculated plants under control conditions; however, the temperature in drought-exposed PGPB-inoculated plants remained unaltered when compared to their control counterparts. Drought progression resulted in a significant increase in temperature in the non-inoculated plants (Figure 5A). Regarding stem water content (WC), slight decreases were observed in plants subjected to moderate and severe drought conditions, irrespective of inoculation status (Figure 5B). However, under extreme drought, significant differences were noted between inoculated and non-inoculated plants, with the latter decreasing to a stem water content of 60%, whereas the former exhibited a less pronounced decrease to 78% stem water content. Concerning stem proline content (Figure 4C), distinct patterns were observed, with non-inoculated plants demonstrating a significant increase in proline even at moderate drought stress, maintaining it at severe drought, and increasing up to seven times the control values at extreme drought stress. Conversely, in inoculated plants, proline increased significantly only when plants were under severe drought stress (comparable levels to their non-inoculated counterparts), increasing only to as high as three times that of the control under extreme drought stress.

### 3.3. Pigment Profile

**Figure 6:**
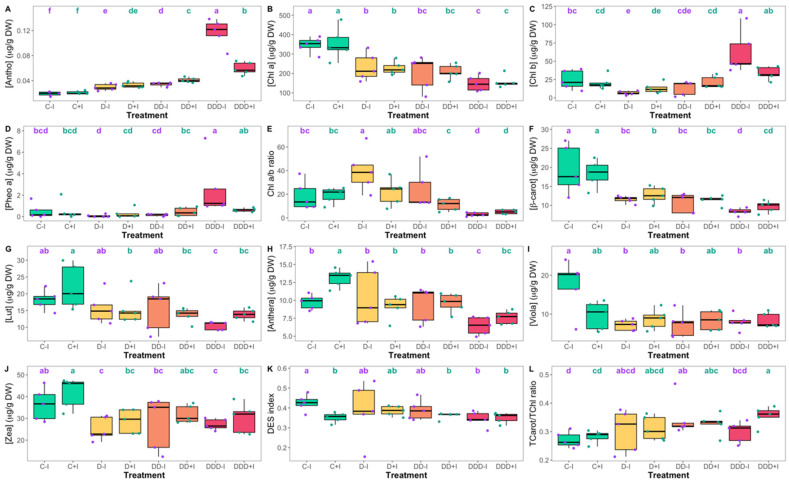
Anthocyanin (**A**), chlorophyll *a* (**B**), chlorophyll *b* (**C**), pheophytin *a* (**D**), chlorophyll *a* to *b* ratio (**E**), β-carotene (**F**), lutein (**G**), antheraxanthin (**H**), violaxanthin (**I**), zeaxanthin (**J**), de-epoxidation state (DES) index (**K**), and total carotenoid to total chlorophyll ratio (**L**) measured in the *Salicornia ramosissima* J. Woods control (C) plants and the individuals exposed to moderate (D), severe (DD), and extreme (DDD) drought conditions with (+I) and without (−I) marine plant growth-promoting bacteria (PGPB) inoculation (N = 5 per treatment, boxplots with average ± standard deviation; letters denote significant differences at *p* < 0.05).

In the present study, anthocyanin accumulation was significantly elevated in plants subjected to any form of drought stress compared to the controls, irrespective of the inoculation status (Figure 6A). Notably, under moderate drought conditions, no discernible difference in anthocyanin levels was observed between the inoculation treatment groups. However, a substantial increase was observed in plants inoculated with PGPB and subjected to severe drought conditions. Under extreme drought conditions, anthocyanin concentrations continued to increase, with non-inoculated plants exhibiting significantly higher levels than their inoculated counterparts. Conversely, the chlorophyll *a* (Figure 6B), chlorophyll *b* (Figure 6C), pheophytin *a* (Figure 6D), chlorophyll *a*/*b* ratio (Figure 6E), and various carotenoids, including β-carotene (Figure 6F), lutein (Figure 6G), antheraxanthin (Figure 6H), violaxanthin (Figure 6I), and zeaxanthin (Figure 6J), exhibited no significant differences between inoculated and non-inoculated plants across all treatments, with the exception of antheraxanthin, which demonstrated significantly higher levels in control plants inoculated with PGPB compared to their non-inoculated counterparts. Comparative analysis with the control groups demonstrated that chlorophyll *a* content (Figure 6B) decreased progressively with increasing drought severity. Chlorophyll *b* levels (Figure 6C) diminished in moderate drought non-inoculated plants relative to their control but increased in both inoculated and non-inoculated plants under drought conditions when compared to their respective controls. Pheophytin *a* (Figure 6D) exhibited a significant increase exclusively in non-inoculated plants subjected to extreme drought conditions. The chlorophyll a/b ratio (Figure 6E) was significantly elevated in non-inoculated plants subjected to moderate drought conditions when compared to their control condition, whereas a notable decrease was observed in the specimens exposed to extreme drought conditions, irrespective of their inoculation status. All drought conditions resulted in a marked reduction in β-carotene content (Figure 6F), with the lowest concentration observed in specimens subjected to extreme drought conditions without PGPB supplementation. Lutein (Figure 6G) and antheraxanthin (Figure 6H) content exhibited distinct patterns between the drought treatments and controls. In non-inoculated plants, both pigments decreased significantly only under the extreme drought conditions. Conversely, inoculated plants exposed to any drought level demonstrated lower levels of these pigments relative to the inoculated control but presented similar values to the non-inoculated controls. Violaxanthin levels (Figure 6I) remained consistent across all inoculated plants, whereas drought-stressed non-inoculated plants exhibited significant reductions compared to their controls, aligning with the levels in inoculated plants. Zeaxanthin concentrations (Figure 6J) were significantly diminished in both inoculated and non-inoculated plants under moderate and extreme drought conditions compared to the controls, and a decreasing trend was observed in plants exposed to severe drought conditions, although not statistically significant. Regarding the de-epoxidation state (DES; Figure 6K), control individuals supplemented with PGPB demonstrated significantly lower values than their non-inoculated counterparts. Under drought conditions, no significant differences were observed between inoculated and non-inoculated plants. Under severe and extreme drought conditions, non-inoculated plants exhibited a significant decrease relative to their control, while inoculated plants maintained consistent DES levels. Finally, the total carotenoids to total chlorophylls ratio (Figure 6L) exhibited an upward trend with increasing drought stress. However, significant differences between inoculated and non-inoculated plants were only observed under the extreme drought condition, with PGPB-inoculated plants showing a notably higher ratio, whereas non-inoculated plants displayed decreased values, similar to their control.

### 3.4. Fatty Acid Profiles

**Figure 7:**
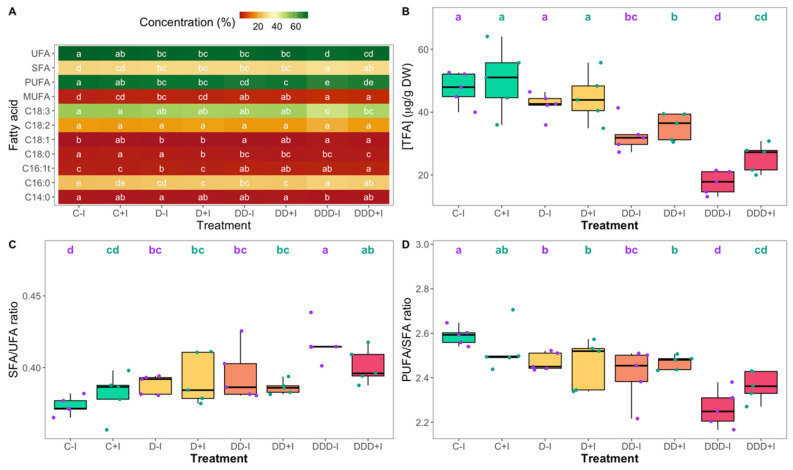
Fatty acids relative concentrations and saturation class ((**A**); UFA—unsaturated fatty acids; SFA—saturated fatty acids; PUFA—polyunsaturated fatty acids; MUFA—monounsaturated fatty acids), total fatty acid content (TFA, (**B**)), SFA/UFA ratio (**C**), and PUFA/SFA ratio (**D**) measured in the *Salicornia ramosissima* J. Woods control (C) plants and the individuals exposed to moderate (D), severe (DD), and extreme (DDD) drought conditions with (+I) and without (−I) marine plant growth-promoting bacteria (PGPB) inoculation (N = 5 per treatment, boxplots with average ± standard deviation; letters denote significant differences at *p* < 0.05).

The fatty acid profile of the *S. ramosissima* photosynthetic stems was examined, focusing on several key fatty acids and their saturation classes (Figure 7A). Myristic acid (C14:0) and linoleic acid (C18:2) levels did not exhibit significant changes, although myristic acid levels demonstrated a minor but statistically significant decrease in non-inoculated plants subjected to extreme drought stress. While both palmitic acid (C16:0) and trans-hexadecenoic acid (C16:1t) increased with increasing drought intensity, the only notable difference between inoculation groups was observed in individuals subjected to moderate drought treatment. For palmitic acid, the consistent and statistically significant increase in both inoculation groups continued to intensify from moderate to extreme drought conditions. In contrast, C16:1t levels in non-inoculated plants increased significantly under moderate drought and plateaued thereafter, whereas, in PGPB-biofertilized plants, a significant increase was initially observed under severe drought and remained similar under extreme drought conditions. Stearic acid (C18:0) exhibited a decreasing trend with increasing drought stress. PGPB-fertilized plants demonstrated significant decreases in stearic acid levels relative to the inoculated control across all drought conditions, with plants under extreme drought reaching the lowest values. In contrast, non-inoculated plants exhibited significant decreases only when subjected to severe and extreme drought treatments, ultimately aligning with the levels observed in the inoculated plants. Oleic acid (C18:1) levels remained significantly unaltered in the inoculated plants compared to the controls, whereas non-inoculated plants exhibited a significant increase in this fatty acid content under severe and extreme drought conditions. Linolenic acid (18:3) levels, when compared to the control, demonstrated a significant decrease only under extreme drought conditions, and no significant differences were observed between inoculation treatments.

No statistically significant differences were observed among the various treatments in the classes of MUFA, PUFA, UFA, and SFA between inoculated and non-inoculated plants. However, similar patterns of fatty acid saturation varied in relation to drought severity in both plant groups; as drought severity increased, UFA decreased, whereas SFA exhibited an increasing trend. Additionally, a significant increase in MUFA and a concomitant decrease in PUFA were observed with increasing drought severity.

Changes in the total fatty acid content (TFA) in the photosynthetic stems were not statistically significant under the different inoculation statuses of the plants (Figure 7B). In both treatments, alterations were observed only under severe and extreme drought stresses, with a tendency for TFA to reach lower levels in non-inoculated plants, although this was not statistically significant.

The saturated to unsaturated fatty acid ratio (SFA/UFA) exhibited a similar trend to TFA in non-inoculated and inoculated plants, with no significant differences between the specimens maintained under control conditions (Figure 7C). An inverse pattern was observed in the polyunsaturated to saturated PUFA/SFA ratio (PUFA/SFA), with significantly lower values in specimens exposed to extreme drought conditions when compared to the levels observed in control plants (Figure 7D).

### 3.5. Oxidative Stress Biomarkers Profiles

**Figure 8:**
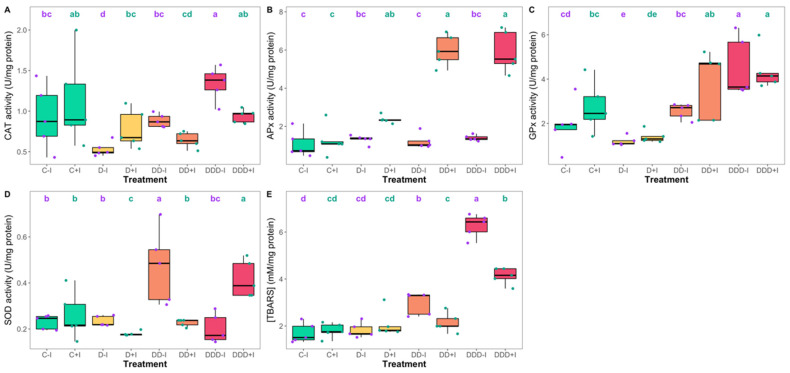
Activities of catalase (CAT, (**A**)), ascorbate peroxidase (APx, (**B**)), guaiacol peroxidase (GPx, (**C**)), superoxide dismutase (SOD, (**D**)) activities, and lipid peroxidation products (TBARS, (**E**)) in the *Salicornia ramosissima* J. Woods control (C) plants and the individuals exposed to moderate (D), severe (DD), and extreme (DDD) drought conditions with (+I) and without (−I) marine plant growth-promoting bacteria (PGPB) inoculation (N = 5 per treatment, boxplots with average ± standard deviation; letters denote significant differences at *p* < 0.05).

Analysis of the catalase (CAT) activity in the plants subjected to various treatments (Figure 8A) revealed significant decreases in non-inoculated plants under moderate drought treatment and in inoculated plants under severe drought conditions. Under extreme drought conditions, no significant differences were observed between the inoculation groups, although the non-inoculated plants exhibited significantly higher activity levels than the respective control. In contrast, no significant alterations were detected in ascorbate peroxidase (APx) activity between the inoculated and non-inoculated control specimens (Figure 8B). However, a significant increasing trend was observed in inoculated plants with increasing drought intensity, whereas non-inoculated plants exhibited no significant changes in response to drought treatments. Regarding guaiacol peroxidase (GPx) activity (Figure 8C), no discernible differences were observed between inoculated and non-inoculated plants within each treatment. GPx activity decreased significantly in plants subjected to moderate drought, but only non-inoculated plants under severe drought treatment demonstrated significantly higher values than their respective controls. For both inoculation treatments, the highest values were recorded in the specimens exposed to extreme drought treatment. With respect to superoxide dismutase (SOD) activity (Figure 8D), only control plants exhibited no significant differences between the inoculation groups. In non-inoculated plants, SOD activity was significantly higher only in the plants subjected to severe drought treatment compared to the control. Conversely, PGPB-biofertilized plants exhibited a slight but significant decrease under moderate drought treatment and a significant increase under extreme conditions, attaining levels similar to those of non-inoculated plants exposed to severe drought. Analysis of the levels of thiobarbituric acid reactive substances (TBARS) (Figure 8E) revealed no differences between the inoculation groups in both the control and moderate drought treatments. Conversely, non-inoculated plants demonstrated a gradually significant increase with increased drought severity, whereas inoculated plants showed a significant increase only under the extreme drought treatment, although still lower than that observed in their non-inoculated counterparts.

### 3.6. Physiological Profiles

**Figure 9:**
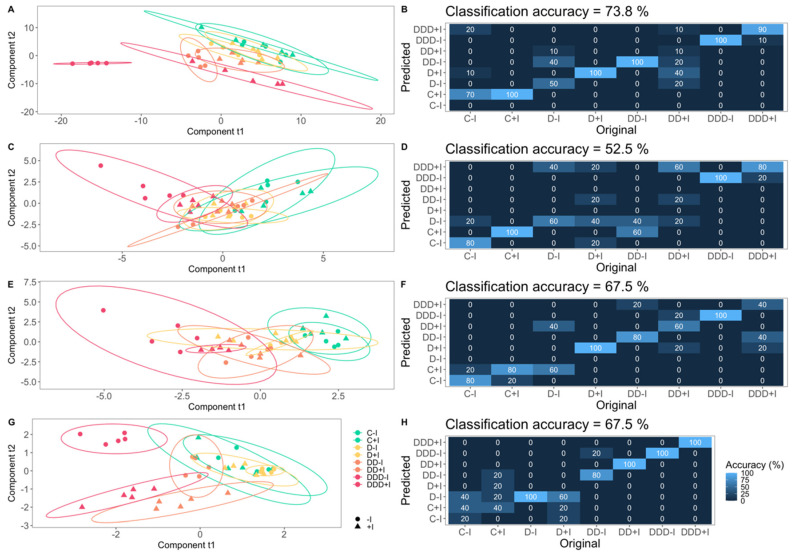
Partial least squares discriminant analysis (PLS-DA) biplots (**A**,**C**,**E**,**G**) and confusion matrix heatmaps (**B**,**D**,**F**,**H**) based on Kautsky curves (**A**,**B**), pigment concentrations (**C**,**D**), fatty acid concentration (**E**,**F**), and oxidative stress biomarker (**G**,**H**) data in the *Salicornia ramosissima* J. Woods control (C) plants and the individuals exposed to moderate (D), severe (DD), and extreme (DDD) drought conditions with (+I) and without (−I) marine plant growth-promoting bacteria (PGPB) inoculation.

A multivariate analysis of the studied physiological traits revealed which aspects of plant metabolism were most impacted by drought and inoculation treatments (see Figure 9). The plants’ photochemical apparatus, evaluated through a multivariate examination of Kautsky curves, showed a distinct separation among treatment groups (Figure 9A,B) with 73.8% classification accuracy. Fatty acid (Figure 9E,F) and oxidative stress (Figure 9G,H) traits displayed similar values (67.5% classification accuracy), suggesting a coordinated response to drought. In contrast, pigment concentration analysis exhibited the lowest classification accuracy (52.5%), with overlapping groups, indicating a potentially diminished effect of the experimental treatments on this variable (Figure 9C,D). Notably, most trait datasets demonstrated significant misclassification between inoculated and non-inoculated samples under milder drought conditions. However, classification accuracy improved with increasing drought stress severity, indicating enhanced resolution in the displayed traits and contrasting sample characteristics.

**Figure 10:**
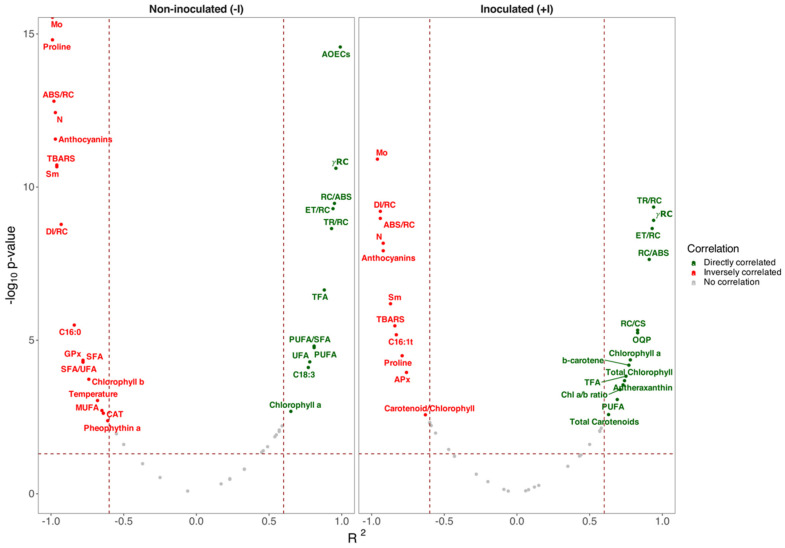
Spearman volcano plots between the photosynthetic stem water content and the photochemical and biochemical traits evaluated in non-inoculated and inoculated *Salicornia ramosissima* J. Woods plants exposed to control and the different drought conditions evaluated (horizontal dashed line indicates the *p* < 0.05 and vertical dashed lines indicate R^2^ = 0.60 (**right**) and R^2^ = −0.60 (**left**) thresholds, delimiting the highly significant and strong correlations).

Upon analyzing the correlations between the plants’ water content and the various physiological traits evaluated, it is evident that 15 variables exhibited similar tendencies in both inoculated and non-inoculated plants. These variables include proline, anthocyanins, chlorophyll *a*, TBARS, PUFA, and TFA concentrations, as well as the photochemical traits M_0_, S_M_, N, γ_RC_, ABS/RC, TR/RC, ET/RC, DI/RC, and RC/ABS (Figure 10). However, it is noteworthy that, with the exception of TR/RC, DI/RC, and chlorophyll *a* concentration, all the remaining common variables demonstrated a decrease in the correlation coefficient absolute value in relation to the plant water content under inoculated conditions. Regarding TR/RC and chlorophyll *a*, these variables exhibited a stronger direct correlation between their values and the plant water content under PGPB inoculation treatment, while DI/RC demonstrated a stronger inverse correlation with the plant water status. It is noteworthy that under non-inoculated conditions, the variables that displayed significant relationships with the plant water content were primarily related to its fatty acid metabolism (C16:0, C18:3, SFA, MUFA, UFA, SFA/UFA, and PUFA/SFA), and to a lesser extent, to the primary (AOECs, chlorophyll *b*, and pheophytin *a*) and oxidative stress metabolism (CAT and GPx). Stem temperature also exhibited a significant correlation with the non-inoculated plants’ water content. Conversely, under PGPB inoculation, all variables that displayed significant relationships with the plant water content were associated with photochemistry (OQP, RC/CS, antheraxanthin, β-carotene, chl a/b ratio, total carotenoids, total chlorophyll, carotenoid/chlorophyll), including the oxidative stress enzyme APx, which is also involved in primary metabolism, and the C16:1t thylakoidal fatty acid.

## 4. Discussion

Anthropogenic climate change significantly impacts plant carbon assimilation by increasing the frequency and severity of short-term droughts, resulting in overall detrimental consequences for plant survival and growth [89,90,91]. The majority of studies investigating the effects of drought on plants have notably excluded highly relevant plant companions and their rhizosphere microbiome, resulting in substantial discrepancies between field results and greenhouse experiments. Consequently, there exists a significant need to examine the impact of these abiotic stressors in the presence of specific microbial communities. This examination should not only evaluate their effects at both physiological and metabolic levels but also explore the potential use of these microbial companions as biotechnological tools for rhizosphere engineering to enhance plant tolerance in sustainable and regenerative cultivation approaches. This consideration is particularly crucial for implementation in bioaugmented cultivation strategies under adverse conditions, especially when utilizing novel cash crop candidates such as *S. ramosissima*.

Exposure to drought resulted in a progressive reduction in inflection points in the Kautsky curve, particularly with increasing stress severity, suggesting detrimental effects on energy transport [92,93,94]. At elevated levels of applied stress, the performance of AOECs in inoculated plants was significantly enhanced. This phenomenon may be attributed to a compensatory mechanism employed by the plants to counteract the observed low energy use efficiency, characterized by diminished trapping and transported energy fluxes [95]. This phenomenon was more pronounced in the bioaugmented plants, demonstrating a statistically significant increase in RC/ABS and ABS/RC within the PSII antennae, accompanied by an elevated S_M_ and a reduced probability of light energy harvesting by chlorophyll molecules under progressively severe drought stress conditions. These findings underscore the significance of the ameliorative function of plant growth-promoting bacteria in mitigating the effects of drought stress by enhancing the plant’s capacity to utilize active reaction centers for heat dissipation, thereby providing protection against elevated temperature and light intensity [96].

Furthermore, the efficacy of PGPB treatment in mitigating stress can be evaluated through the assessment of relative energy dissipation, which serves as an indicator of inefficiency in the photochemical utilization of energy. This phenomenon was evidenced by the observed temperature increase in non-inoculated plants. The elevation in plant temperature is frequently associated with stomatal closure, which subsequently leads to a reduction in evaporative cooling [97,98,99]. Conversely, temperature exhibited greater stability in inoculated plants, indicating enhanced thermoregulation. Mild drought stress in PGPB-inoculated plants facilitated increased energy flux of electron transport and decreased dissipated energy, which was further augmented by a higher quantity and efficiency of reaction centers, as evidenced by RC/ABS and γ_RC_. The elevated DI/CS in non-inoculated plants may suggest heat dissipation or photorespiration energy-quenching defense mechanisms [100]. Inoculation with ACC deaminase-producing bacteria may have been responsible for the observed stress alleviation. Ethylene levels increase in plant tissues under abiotic or biotic stress and negatively impact plant growth [101]. ACC deaminase-producing bacteria, through the cleavage of ACC into α-ketobutyrate and ammonia, can mitigate the deleterious effects of ethylene on plant growth under drought stress conditions [102,103,104]. Inoculation with such bacteria has been demonstrated to be an efficacious cultivation strategy for enhancing abiotic stress tolerance [105]. It has also been demonstrated to enhance photosynthetic efficiency, which is most likely attributable to reduced photochemical stress and, consequently, increased energy use efficiency [106,107]. While the precise mechanisms through which ACC deaminase-producing bacteria enhance photosynthetic processes remain incompletely understood, previous studies have demonstrated the influence of various environmental stress factors on the concentration of photosynthetic pigments [108]. This is related to the results presented herein.

Although the stem temperatures for the inoculated control plants exhibited higher values, they were not correlated with drought severity, consistently remaining lower than those in non-inoculated plants, which indicates an enhancement in stomatal regulation [27]. The water content (WC) trend under extreme drought exhibited significant differences; it decreased markedly to 59% WC for the non-inoculated plants, while for the inoculated plants, the decrease was less pronounced at 77%. Proline accumulation increased seven-fold in the non-inoculated plants under extreme drought stress, serving as a mechanism for enhancing osmoregulation and mitigating the effects of water loss. In contrast, the inoculated plants demonstrated only a three-fold increase in proline accumulation under identical conditions. This observation substantiates the presence of increased yet appropriate osmoprotection via PGPB actions in the inoculated plants and aligns with previous findings on bioaugmented plants [109,110]. Inoculation with PGPB may potentially mitigate water loss and provide a viable strategy for maintaining agricultural productivity under increasingly arid conditions. This approach appears particularly applicable to the cultivation of halophytes, such as *S. ramosissima*, in regions characterized by limited water availability [48,49].

Drought stress reduced chlorophyll *a* levels in both inoculated and non-inoculated plants. This represents an adaptive response to excessive irradiance under drought stress [111]. Under extreme drought conditions, only non-inoculated plants exhibited chlorophyll degradation, as evidenced by elevated levels of phaeophytin *a*. Conversely, in PGPB-inoculated plants, chlorophyll content was maintained without significant accumulation of pheophytin *a* under extreme conditions, which substantiates the protective role of PGPB inoculation on photosynthetic pigments [112,113]. While carotenoid levels decreased in inoculated and non-inoculated plants under extreme drought conditions, anthocyanins, pigments with ROS-scavenging properties [114], increased in proportion to drought severity, particularly in non-inoculated plants subjected to extreme drought. The observed increase in anthocyanin levels, concomitant with the decrease in DES, suggests that the xanthophyll cycle was not significantly activated, indicating that the plants may have shifted towards anthocyanin-mediated photoprotection as a mechanism to mitigate oxidative stress [115]. Despite the observed increase in dissipated energy flux (DI/RC), the absence of xanthophyll cycle engagement suggests the potential activation of alternative energy-quenching mechanisms, such as heat dissipation or photorespiration [15,116].

Collectively, these factors influence plant responses to oxidative stress. In general, drought stress enhances antioxidant enzyme activity in both inoculated and non-inoculated plants subjected to moderate drought. Nevertheless, only in inoculated plants was high antioxidant activity maintained under extreme drought stress, whereas in non-inoculated plants, antioxidant systems were overwhelmed; enzyme activity was diminished and lipid peroxidation was elevated. Proline also serves important functions in ROS-scavenging and thermostabilizing enzymes [110]. Under severe drought conditions characterized by increased ROS production and water loss, inoculated plants exhibited elevated antioxidant enzyme activities, while proline-mediated osmoregulation functioned synergistically to mitigate stress levels and maintain intact metabolic activity [117,118,119]. This observation was further corroborated by the reduced lipid peroxidation in the inoculated plants exposed to extreme drought conditions, which is indicative of the alleviation of oxidative stress. Additionally, fatty acid remodeling provides a means for osmoregulation through a balanced increase in saturation, with the increase in MUFA and decrease in PUFA contributing to membrane stability by reducing membrane fluidity during osmotic stress. This was evidenced by the increase in SFA/UFA ratio and the decrease in PUFA/SFA ratio under drought conditions, particularly in non-inoculated plants. Inoculated plants exhibited a more balanced fatty acid profile, which indicates better membrane function and stress tolerance, similar to that observed in halophytes for the maintenance of osmotic stability [19,120,121].

The present study demonstrates the significant potential of PGPB inoculation for mitigating the impacts of drought stress on *S. ramosissima*, a promising halophyte species, in sustainable agriculture. These findings necessitate further investigation regarding scalability and feasibility in practical application settings. While the results are promising, they also highlight several challenges that must be addressed when scaling up PGPB inoculation for extensive agricultural use. These challenges include the selection of appropriate PGPB strains for reproducibility in various soil types, environmental conditions, and other crops, the refinement of inoculation methods concerning seed coating and soil drenching, and ensuring the long-term survival of the inoculated bacteria in field conditions. Furthermore, economic considerations, such as production and application costs of PGPB inoculants, should be evaluated to ensure economic viability for agricultural practitioners.

Given the limited duration and controlled conditions of the present study, further research is necessary to evaluate the effect of such treatments on plant growth, yield, and stress tolerance under field conditions to assess sustainability and long-term performance. Moreover, investigating the inoculation effects of PGPB on various Salicornia species or other halophytes will provide broader applications for diverse saline agricultural systems. Additional research should also aim to elucidate the complex interplays between PGPB, plants, and other rhizosphere microorganisms. Understanding these interactions can inform the development of optimal inoculation strategies for PGPB and maximize their efficacy. Fundamental research into the molecular mechanisms underlying the observed beneficial effects of PGPB, such as gene expression involved in stress response and osmoregulation, may be crucial in developing more specific and efficient bioaugmentation strategies. Thus, addressing these limitations and future research directions may enable the full potential of PGPB inoculation as a means of improving drought tolerance in *S. ramosissima* and other halophytes for their eventual successful application in sustainable and regenerative agricultural practices.

The joint analysis of metabolic traits and datasets presented herein substantiates the contribution of PGPB in mitigating drought stress. Photochemical, fatty acid composition, and oxidative biomarkers revealed the underlying coordinated mechanism for improved drought tolerance and, consequently, allowed effective phenotypic discrimination among treatments addressed in this study. Indeed, the volcano plots correlation analysis reinforced this hypothesis, elucidating a shift in the drought influence on water content from fatty acid metabolism in non-inoculated plants subjected to increasing drought conditions to the primary metabolism in their PGPB-inoculated counterparts. Furthermore, this multivariate approach provided a comprehensive explanation of which metabolic traits were most affected by drought severity and PGPB inoculation, highlighting the potential use of such datasets as phenotyping tools under drought stress and/or bioaugmentation regimes on *S. ramosissima*, as observed in previous studies addressing other abiotic stresses [16,62,122]. Furthermore, our findings provide insights not only into the mechanisms underlying drought stress tolerance but also into the potential impacts on nutritional characteristics of cash crops.

## 5. Conclusions

The cultivation of potential drought-tolerant cash crops, such as *S. ramosissima*, is becoming increasingly crucial as climate change exacerbates drought events, leading to growing concerns over water scarcity. This has prompted research into novel agricultural cultivars that can thrive in arid conditions, with halophytes emerging as suitable candidates. The present study is of particular significance because it demonstrates that, despite this halophyte’s characteristic high tolerance to drought stress, PGPB-inoculated plants were able to retain more water than their non-inoculated counterparts, highlighting a potential solution to the pressing issue of water scarcity. Native to salt marshes, the photochemical adaptations of *S. ramosissima* involve active osmoregulation, energy redistribution, and membrane remodeling, which enable the maintenance of highly functional photosystems, thereby ensuring primary productivity, even under severe drought conditions. This intrinsic tolerance, in conjunction with the extraordinary added benefits that marine PGPB consortia could provide by further enhancing their resilience, underscores their potential as a viable alternative to traditional crops under adverse conditions. Notably, similar physiological responses in halophytes warrant further investigation with regard to other halophyte species and may lead to the elucidation of conserved mechanisms underlying adaptation among species. This would provide a fundamental basis for developing truly sustainable and regenerative methods of cultivation, leveraging the inherent resilience of halophytes together with bioaugmentation as an eco-friendly biotechnological tool to ensure food security under climate change.

## Figures and Tables

**Table 1:** Photochemical variables determined through the OJIP test and evaluated in this study.

Variable	Description
M_o_	The net rate of PS II RC closure
OQP	Corresponds to the oxidized quinone pool size available for reduction and is a function of the area above the Kautsky plot
S_M_	Energy needed to close all reaction centers
N	Quinone redox turnover rate.
γRC	The probability that a chlorophyll *a* molecule functions as a light-harvesting reaction center.
ABS/RC	Absorbed energy flux per reaction center.
TR/RC	Trapped energy flux per reaction center.
ET/RC	Electron transport energy flux per reaction center.
DI/RC	Dissipated energy flux per reaction center.
RC/ABS	Reaction center density within the PS II antennae.
RC/CS	The number of available reaction centers per leaf cross-section.

## Data Availability

Data will be made available on request.

## Supplementary Materials

The following supporting information can be downloaded at: https://www.mdpi.com/article/10.3390/plants14081227/s1. Table S1: Bacterial strains and their plant growth-promoting properties.
