# Supplementary material for: Enhancing Drought Tolerance in Salicornia ramosissima Through Biofertilization with Marine Plant Growth-Promoting Bacteria (PGPB)
Source: Plants (Basel). 2025 Apr 16;14(8):1227. doi: 10.3390/plants14081227 (PMC12030189; doi:10.3390/plants14081227)
Supplement: Supplementary file 1 [file plants-14-01227-s001.zip › plants-3563840-supplementary.pdf]

Supplemental Information

Table S1. Bacterial strains and their plant growth-promoting properties.

			Plant-Growth Promoting Traits					
**Bacterial Strains**	**Sampling Site**	**Limit Salt Tolerance (mM)**	**P-Solubilization**	**Siderophore**	**ACC Deaminase (nm mg^{-1} h^{-1})**	**IAA (µg mL^{-1})**	**N-Fixation**	**EPS (OD_{540})**
Bacillus aryabhattai SP20	Aveiro coastal lagoon, Portugal	856	+	+	−	−	−	0.80 ± 0.01
Stenotrophomonas rhizophila EH7	Aveiro coastal lagoon, Portugal	856	+	+	−	15,02 ± 0.31	+	−
Pseudomonas oryzihabitans RL18	Tagus Estuary, Portugal	1711	+	+	+* (26.9 ± 14.13)	39.55 ± 1.01	−	0.40 ± 0.01
Salinicola endophyticus EL13	Tagus Estuary, Portugal	1711	+	+	+*	71.35 ± 6.86	−	0.54 ± 0.01

+ positive; − negative; +* visible growth on solid DF +ACC medium.
